# Fair foundations: ensuring epistemic justice in neurodiversity research and practice

**DOI:** 10.3389/fpsyg.2026.1826790

**Published:** 2026-08-13

**Authors:** Ludmila Praslova

**Affiliations:** Graduate Organizational Psychology, Vanguard University of Southern California, Costa Mesa, CA, United States

**Keywords:** community-engaged research, epistemic justice, neurodiversity, neuroinclusion, workplace research

## Abstract

The participation of neurodivergent employees can meaningfully enhance organizational effectiveness - but only when the research and practices that shape workplaces are built on accurate, community-grounded knowledge. This paper develops a perspective that epistemic justice, the recognition of individuals and communities as experts on their own experience, is both an ethical practice and a prerequisite for beneficial neurodiversity research and implementation. Drawing on an epistemic injustice foundational framework that includes testimonial and hermeneutical injustice, this paper extends the concept to propose appropriative injustice, defined as the extraction of community knowledge without attribution or benefit. It further explores how testimonial, hermeneutical, and appropriative justice could operate across the full research-to-practice pipeline and offers concrete, stage-by-stage guidance for graduate educators, researchers, journal editors, practitioners, and organizational leaders who want their work to be both rigorous and just.

## Introduction

Research on neurodiversity in the workplace, while still a young field, has expanded rapidly since 2019 (Rollnik-Sadowska and Grabińska, 2024). It is driven by both academic curiosity and pragmatic organizational considerations. Understanding the neurodivergent experience at work and creating environments that welcome neurodiversity can help improve work for everyone (Praslova, 2024).

The development of the neurodiversity paradigm within the Autistic community in the 1990s was a movement against the dominant medical model that presented neurodivergent differences as deficits requiring correction, not as differences that might allow people to thrive in some environments while other environments might be painful or conducive to their bullying and mistreatment (Botha et al., 2024; Praslova, 2024).

From its inception, the neurodiversity paradigm also functioned as a moral rejection of a clinical and research tradition that had pathologized, excluded, infantilized, and denied neurodivergent people epistemic authority and self-determination (Silberman, 2015; Chapman, 2023; Walker, 2021; Botha et al., 2024). This paper builds on the ethical tradition of the neurodiversity movement and extends it to the research and practice infrastructure that still reproduces documented harms to neurodivergent communities (Botha et al., 2025; Zaks, 2025). These harms call for swift action in establishing safeguards. This paper proposes such safeguards based on the moral principles of biomedical ethics: respect for autonomy, non-maleficence, beneficence, and justice (Beauchamp and Childress, 2019) and the broader ethics of honoring human dignity (Hicks, 2011). In research and practice involving vulnerable or underrepresented populations, commitment to these principles is often operationalized as participation and input of these groups in research (Israel et al., 1998; Nicolaidis et al., 2019). Foundationally, the Belmont Report’s justice principle that research should not exploit vulnerable populations for the benefit of others also supports this paper’s normative argument (National Commission for the Protection of Human Subjects of Biomedical and Behavioral Research, 1979).

## Neurodiversity: the nature and culture

Neurodiversity refers to the full, natural range of human neurological variation. Importantly, which segments of this variation might be considered “neurotypical” is a cultural moving target: the profiles considered “ideal” or “normal” among Viking raiders would be different from modern-day Canadians. People whose neurobiology diverges from prevailing norms are referred to as *neurodivergent*, a term that may refer to differences in emotional experience, attention, sensory processing, communication, and cognition. It may include the innate *neurominority* developmental, social, and learning profiles often framed as disorders and subject to devaluation and discrimination (autism, ADHD, dyslexia) and a broader range of differences such as anxiety, the sequelae of brain trauma, or giftedness (Pasarín-Lavín et al., 2024; Praslova, 2024, 2025; Walker, 2021).

Media and even some academics often use the terms neurodivergent and neurodiversity in ways inconsistent with their meaning within neurodivergent communities. The term neurodivergent was coined by activist Kassiane Asasumasu as broad and not limited to developmental differences (Walker, 2021). Neurodiversity is a collective property of human groups comprising multiple neurotypes, and not an individual characteristic (Praslova, 2025; Walker, 2021).

Neurodivergent individuals are a significant segment of the workforce as well as job-seekers, and understanding their experiences, contributions, and needs matters for building workplaces that work for everyone. Yet there is a rarely named tension at the heart of neurodiversity at work research: the quality of the knowledge we generate depends on the extent to which the research practices epistemic justice toward neurodivergent communities. It depends on whether neurodivergent individuals and communities are seen as active shapers of knowledge about them, or simply data points for others to extract and interpret. When neurodivergent experiences are treated as data points rather than expert testimony and authentic narratives, the resulting studies may ask and answer the wrong questions and produce suggestions that either fail to enhance neurodivergent experience and work in general, or even cause harm. In addition, when uncredited knowledge is extracted from communities with less societal power and used for the benefit of those with more power, the opportunity gap deepens and the community experiences cumulative harm (DiPrete and Eirich, 2006; Smith, 2012).

## Neurodiversity research: breakthroughs and biases

Epistemically just neurodiversity science, on the other hand, is ethical as well as methodologically sound and practically useful (Botha et al., 2025; Zaks, 2025). It is also essential for preventing special interests from taking advantage of vulnerable populations (e.g., Bottema-Beutel et al., 2026).

Knowledge built with communities is likely to be more accurate than knowledge built about them. For example, recent studies that included at least one autistic research team member have helped to debunk the misconceptions about autistic ethical “deficits” while providing important insights for helping behavior in organizations. Findings suggest that autistic employees might be less prone to the classic bystander effect, and thus more likely to intervene in workplace situations that call for identifying and reporting problematic practices (Hartman et al., 2023; Hartman and Hartman, 2024). Autistic adults report lower moral disengagement and are less prone to using self-serving rationalizations for unethical behavior than non-autistic employees (Hartman and Hartman, 2024).

Mottron’s (2011) long-running program of participatory research, including many studies conducted with Michelle Dawson, has debunked many misconceptions about autism and intelligence. It has repeatedly demonstrated that when studies are designed to respect autistic processing styles, autistic participants often outperform non-autistic controls on a range of pattern detection, attention to detail, and other cognitive tasks (Mottron et al., 2006; Mottron, 2011). Other collaborative work strengthens the point that when neurodivergent people are treated as knowers and experts on their own experiences, strengths, and performance needs, and science gains access to insights that less participatory models miss (Pellicano and den Houting, 2022; Pickard et al., 2022).

Unfortunately, such participatory work is not yet common. Moreover, the idea that autistic people are not capable knowers is still prevalent among autism researchers. Botha and Cage (2022) documented that in a survey of 195 autism researchers, 60% of responses included dehumanizing, objectifying, or stigmatizing views about autistic people. Most studies conducted by allistic autism researchers ignore the harms to the autistic community and fail to control for them (Dawson and Fletcher-Watson, 2022). One drastic example is research on Applied Behavior Analysis (ABA) with autistic populations. Autistic-led and community-attuned research supports the concerns about ABA potentially causing excessive levels of compliance that may endanger autistic people (Sandoval-Norton and Shkedy, 2019), as well as PTSD from the “therapy” itself (Kupferstein, 2018). However, this community-informed work is vastly outnumbered by a large volume of publications supportive of ABA therapies – and the overwhelming majority of authors writing in support of ABA hold undisclosed conflicts of interest (COI) (Bottema-Beutel et al., 2026). In a sample of intervention-focused articles published in eight ABA journals, 78% of researchers benefited financially from the ABA industry. Moreover, 93% of them made false statements denying the COI.

A big-picture analysis suggests that, in its current state, autism research remains dominated by neuronormative bias and the extractive interests of the Autism Industrial Complex (Broderick and Roscigno, 2021). Similarly, Snellman et al. (2023) document pharmaceutical COI distorting the ADHD treatment evidence base, while Sismondo (2021) exposes a broader epistemic corruption in the pharmaceutical research industry.

This prevalence of bias and corruption places autistic autism researchers and neurodivergent neurodiversity researchers in the position of trying to protect our communities from epistemic injustice while simultaneously being targets of that injustice in our own fields, and of having to occupy a taxing role as academics, activists, and advocates at once (Botha, 2021). Such multifaceted epistemic experience of the author also informs this paper.

## Epistemic injustice framework and neurodiversity research

Fricker (2007) explains that epistemic injustice occurs when a person or a group is wronged “in their capacity as a knower.” The dynamics of prejudice, power, or structural entrenchment privilege some knowers while undermining the ability of others to contribute to knowledge or have their knowledge recognized as credible. An example of epistemic injustice may be a paper on autism and employment that fails to acknowledge the controversial nature of constructs imposed by clinicians but rejected by many in the Autistic cultural community, such as functioning labels (e.g., high vs. low functioning) or the deficit framing (Alvares et al., 2020; Keates et al., 2025). Similarly, researchers may ignore the critique of framing dyslexia as a “reading disability” by dyslexic scholars who argue that dyslexia reflects a different cognitive architecture, associated with powerful spatial reasoning and big-picture thinking that is disadvantaged by text-heavy environments (Eide and Eide, 2011).

Researchers may use sophisticated statistical analyses while treating contested academic categorizations as objective facts and discounting decades of community thought leadership that challenges these frameworks as stigmatizing and inaccurate (Botha, 2021; Dark, 2024). Quantitative rigor cannot compensate for the fundamental injustice of imposing rejected frameworks on a marginalized community that has developed its own self-understanding and self-definitions. In what follows, I draw on Fricker’s (2007) model of epistemic injustice while extending it to name an additional form of injustice that occurs in neurodiversity research and practice.

Fricker (2007) identified two primary forms of epistemic injustice. *Testimonial* injustice arises when a person’s knowledge claims are systematically discounted because of prejudice about who they are. In other words, people are not seen as capable of accurately understanding and/or relaying their own experience. Testimonial injustice may also arise, for example, when neurodivergent employees describe sensory overwhelm or masking fatigue as resulting from environmental stress, but their reports are filtered through an individual deficit lens and recast as symptom description. The employees’ interpretation of their experience is seen as unreliable, and they become subjects of interpretation rather than authors of understanding. They are denied the first-person authority routinely granted to neurotypical workers.

*Hermeneutical* injustice occurs when the dominant conceptual frameworks fail to accurately reflect or even actively distort the experiences of particular groups, leaving them without the vocabulary to make sense of, or be understood in, their own lives. For example, referring to non-speaking autistic people as low-functioning ignores the fact that the lack of spoken communication has not precluded individuals from earning graduate degrees or publishing books (e.g., Chan, 2025; Mukhopadhyay, 2008). The insistence of the clinical community, until 2013, that autism and ADHD cannot coexist (Leitner, 2014; Young et al., 2020), illegitimized the lived experience of many who now identify as AuDHD.

In addition to the two forms of epistemic injustice identified by Fricker (2007), I observed a third form relevant to research and its application: appropriative injustice.

*Appropriative injustice* describes what happens when the *authorship and labor involved in* neurodiversity work are systematically misaligned with *recognition and rewards*. This may occur in two stages, in which the second is used to cover up the harm perpetrated by the first, while, in fact, building on and expanding that harm.

First, neurodivergent communities develop ideas and language, such as the neurodiversity paradigm or neurodivergent accounts of masking, that are then incorporated into academic articles, consulting products, and organizational initiatives with little or no credit, co-authorship, or tangible benefit flowing back to those communities. Sometimes, the knowledge is misunderstood, misinterpreted, or misrepresented to suit the researcher’s or commercial agenda. Second, in some cases, neurodivergent people are invited to serve as reviewers, advisors, or “experts by experience” and are asked to fix and polish this work so it can be published, funded, or implemented, usually without pay, authorship, credit, or influence over final decisions. This, in a way, legitimizes the appropriation as community-reviewed while denying that it is community-originated.

Accounts from the broader literature on social bias document related phenomena of epistemic oppression and exploitation (Dotson, 2012; Berenstain, 2016). Similarly to epistemic exploitation, appropriative injustice may involve emotional harm and a demand for unpaid labor (Berenstain, 2016). However, it more directly centers on knowledge appropriation and on how authorship, credit, pay, and career advantages are redistributed away from neurodivergent communities and individuals and toward institutional and individual actors who repackage their insights, echoing concerns about extractive and tokenistic research practices (Smith, 2012; Kadodia and Krueger, 2026). The incentives, such as citation counts, promotion, grants, consulting revenues, and career opportunities, flow to those who appropriate community knowledge rather than to those who created it. Appropriative injustice perpetuates a pattern of extraction that maintains the exclusion and discounting of marginalized communities by reinforcing the assumption that the community’s knowledge is there for the taking. This also undermines the community’s trust in science and compromises the integrity of the scholarly record.

All these forms of injustice: testimonial, hermeneutical, and appropriative, are not inevitable features of neurodiversity research, but rather correctable, often implicit tendencies that can be reduced with appropriate guardrails. Moreover, the corrective is both an ethical improvement and methodological strengthening. Community-engaged quantitative research produces more accurate and actionable findings (Torres Rodríguez et al., 2023). Meaningful participation of the autistic community in autism research can improve science and help mend the harm inflicted by epistemic injustice (Botha, 2021; Fletcher-Watson et al., 2019; Nicolaidis et al., 2019).

Consider interventions designed to teach autistic individuals interview and workplace social skills, teaching organizational skills utilizing assistive technology, or behavioral training to make autistic people better tolerate interruption (Chen et al., 2015; Kumazaki et al., 2019; McKenna et al., 2020). Scott et al.’s (2019) review of 36 intervention-based studies casts much doubt on the effectiveness of individual-focused interventions. While these might increase vocational and executive functioning skills, participants largely remained unemployed because the organizational environment and coworker biases were never addressed. If autistic people had an opportunity to shape study designs, they likely would have steered the effort toward the more promising environmental interventions.

To build research and practice on just foundations, an ethical upgrade is needed to epistemic infrastructure in the research-to-practice pipeline. Drawing on traditions of addressing and preventing harm in biomedical (Beauchamp and Childress, 2019) and public health ethics (Israel et al., 1998), the remainder of this paper advances a normative operationalization of epistemic justice in neurodiversity research and professional practice. Within these traditions, addressing and preventing harm to humans or communities constitutes a foundational ethical commitment. Some of the suggested guardrails are supported by empirical research. Where such research is not available, propositions stem from the moral principles of biomedical ethics: respect for autonomy, non-maleficence, beneficence, and justice (Beauchamp and Childress, 2019), as well as the principle of community participation as a foundational requirement for ethical research and practice with marginalized populations (Fletcher-Watson et al., 2019; Israel et al., 1998; Nicolaidis et al., 2019; Thomas, 2024; Wilson, 2008).

Proposed principles are not meant to replace the existing methods of ensuring research quality or specific disciplinary and institutional procedures. They are meant to add guardrails and tools to support the just treatment of neurominority populations.

## Embedding epistemic justice across the research-to-practice pipeline

### Graduate training and assumption formation

The epistemic habits that shape research begin in graduate education, if not earlier. Programs in psychology, organizational psychology, human resources, and management often transmit, implicitly and explicitly, a medical model of neurodivergence: traits as deficits, accommodation as charity, success as conformity to neurotypical norms (Praslova, 2024). Students absorb these assumptions before they have designed a single study.

Reorienting graduate training does not require a full curriculum redesign. Several targeted changes, as appropriate within specific fields and institutional contexts, can make a meaningful difference:

- Assign scholarship authored by neurodivergent researchers as required reading, not supplementary material. Ensuring that students encounter neurodivergent perspectives as foundational contributions to the field, rather than optional add-ons, helps model epistemic justice.
- Another way to model epistemic justice is inviting neurodivergent practitioners, advocates, and scholars as course guest speakers and thesis committee members—not to “represent” their community but to contribute expertise in their areas of scholarship.
- Include community-engaged research methods in methods courses alongside experimental and survey designs. Participatory action research, community-based participatory research, and co-design approaches all have well-developed methodological literatures (Fletcher-Watson et al., 2019; Dark, 2024; Nicolaidis et al., 2019).
- Help students develop a habit of distinguishing between researcher interpretation and community self-understanding, and treating misalignments between them as important data rather than noise to be explained away in favor of the researcher.

### Research design and question formulation

The most consequential epistemic decisions in a study are made before data collection begins. Research questions encode assumptions about who has problems, who has answers, and whose interests the findings should serve. Epistemic justice at this stage can be promoted by asking:

- Does this question emerge from community priorities or from academic trend-following?
- Does the theoretical framework reflect how neurodivergent people understand their own experiences?
- Are neurodivergent people positioned as co-investigators, not just participants?
- Will the findings be accessible and useful to the communities whose lives they describe?

Community advisory groups composed of neurodivergent individuals with relevant experience, not treated as free labor, can help researchers pressure-test their framing before committing to a design (Fletcher-Watson et al., 2019; Dark, 2024; Nicolaidis et al., 2019). A 2-h advisory consultation at the design stage can prevent years of research built on miscalibrated assumptions.

Consider the difference between two research questions: “How do successful employees with Tourette’s minimize the impact of tics on workplace performance?” vs. “What workplace conditions allow employees with Tourette’s to perform at their best?” Same workplace. Same problem. The community involvement in asking research questions can help direct efforts toward improving people’s lives.

Researchers should also attend carefully to language. Community-preferred terminology (“autistic person” rather than “person with autism” for many in the autistic community; “neurodivergent” rather than “atypical”; “neurotypical” rather than “normal”), with correction for individual preferences in direct interactions, signals epistemic respect (Keating et al., 2023; Smith et al., 2023).

### Peer review and publication

Peer review functions as the field’s epistemic gatekeeper, and the standards applied at this stage shape what counts as knowledge and whose knowledge counts (Fricker, 2007). This gatekeeping is not neutral: evaluation criteria, reviewer composition, and editorial culture collectively determine which frameworks are treated as rigorous and which are dismissed as insufficiently empirical (Botha, 2021). In neurodiversity research specifically, these dynamics have demonstrably disadvantaged community perspectives while privileging frameworks that pathologize neurodivergent populations (Dawson and Fletcher-Watson, 2022; Botha and Cage, 2022). The problem is compounded by undisclosed conflicts of interest (Bottema-Beutel et al., 2026). Several structural adjustments can build epistemic accountability into the review process: Journals considering neurodiversity scholarship can strengthen epistemic justice by adding its criteria to reviewer guidelines: Does the paper honor community self-understanding? Does it cite and credit community-generated frameworks? Does its framing honor dignity (Hicks, 2011) and wellbeing (Beauchamp and Childress, 2019) of the population studied?

- Reviewers should be prepared to recognize methodologically rigorous work that nonetheless commits epistemic injustice, and advance work that integrates community epistemology without sacrificing analytical rigor. These are not mutually exclusive expectations.
- A practical checklist for reviewers and editors might ask: Does this paper engage with scholarship produced by members of the focal community? Does it use language the community has endorsed? Does it avoid framing neurodivergent traits as problems without also examining the organizational conditions?
- Editorial boards of relevant publications should include neurodivergent researchers, not tokenistically but in sufficient numbers to influence review culture and reviewer selection.
- Currently, reviewers who raise concerns about respecting the community epistemology, especially those with lived experience of the population studied, might be asked to substantially educate authors on community frameworks and, de-facto, help correct epistemic injustice. This is a form of epistemic labor that is systematically uncompensated in marginalized communities (Berenstain, 2016). To support justice, I suggest that journals carefully consider whether placing so much of the revision burden on neurominority or neurodivergnet reviewers is fair. Placing a reviewer, especially when a reviewer is a member of the population against which the epistemic violence is being committed, in a position of providing uncredited labor of correcting injustice, represents a uniquely problematic form of extraction. In effect, what occurs in such cases is appropriating reviewers’ knowledge about epistemic justice and, in some cases, their painful lived experiences as members of marginalized groups, to drastically improve the credited authors’ submission. A high moral cost is levied when reviewers’ lived experience and labor become part of the final work, while authors claim credit for insights they neither originally possessed nor could have independently developed.

To reduce appropriative injustice in research, epistemic justice has to be treated as a basic quality standard, not an optional extra (Bottema-Beutel et al., 2026; Fricker, 2007). That means building neurodivergent leadership into editorial structures, updating editorial and reviewer guidelines to check for fair credit and accurate use of community frameworks (Fletcher-Watson et al., 2019; Israel et al., 1998; Nicolaidis et al., 2019), and recognizing the extent of the epistemic labor often expected of neurominority or other marginalized reviewers (Berenstain, 2016; Botha, 2021). Fairer research, in turn, is likely to support fairer practice (Torres Rodríguez et al., 2023).

### Organizational research and practice implementation

The transition from published research to organizational policy introduces its own epistemic risks. Practitioners often work from simplified summaries of findings, and the nuance that community-engaged research introduces is frequently the first casualty of simplification and implementation. Several practices can help preserve epistemic integrity at this stage:

- Involve neurodivergent employees in designing, piloting, and evaluating any neurodiversity initiative—not as participants in a program designed for them, but as consultants and co-designers with genuine input into outcomes (Praslova, 2024).
- Distinguish between interventions that reduce barriers (genuinely useful) and programs that require neurodivergent employees to mask and perform neurotypicality more skillfully (often harmful and counterproductive). Barrier-reduction approaches address the organizational conditions that impede neurodivergent performance, including harsh sensory environments, narrow communication norms and subjective evaluation criteria and are consistent with the social model of disability (Oliver, 2013; Scott et al., 2019). On the other hand, programs that train neurodivergent employees to mask demand the cognitive and emotional load of suppressing a natural neurological expression (Hull et al., 2021), may lead to burnout and poor mental health (Raymaker et al., 2020), and center on others’ comfort rather than neurodivergent needs. In essence, masking-focused interventions can produce surface-level compliance while deepening harm.
- Establish internal feedback mechanisms through which neurodivergent employees can report whether initiatives are working, without fear of being labeled “difficult” or “resistant.”
- When commissioning external research, require community engagement plans as a standard deliverable alongside methodological protocols. Ask vendors to specify how neurodivergent community members were involved in question formulation and interpretation.

Organizations that genuinely want to benefit from neurodivergent contributions to problem-solving, pattern recognition, systems thinking, and creativity must build environments in which neurodivergent employees can contribute authentically rather than expend energy managing their presentation. Epistemic justice in the form of participatory research (e.g., Nicolaides, 2019) can help us determine what those environments actually look like.

A note of caution is warranted here. A growing genre of organizational literature makes the “business case” for neurodiversity, quantifying the cognitive return on inclusion, cataloging neurodivergent strengths as competitive assets, positioning accommodation as investment (Praslova, 2024). This framing, however well-intentioned, carries its own epistemic risk. When inclusion is justified primarily through productivity metrics, it implicitly conditions the dignity of neurodivergent employees on their demonstrable organizational utility. It also subtly reframes the question from “what do people need to thrive?” to “what can we extract?” The latter is precisely the extractive logic that community-engaged research is designed to correct. Organizational leaders should be skeptical of frameworks that require neurodivergent employees to prove their worth in neurotypical terms before being afforded basic dignity and structural support.

### Dissemination and community benefit

Research that communities cannot access, understand, or use becomes a form of epistemic withholding even when it was produced with good intentions. Closing the community benefit loop could mean:

- Sharing findings in accessible formats with the communities that contributed to the research via plain-language summaries, community presentations, or practitioner guides, not only journal articles behind paywalls.
- Properly attributing community-developed frameworks and concepts in all disseminated materials, including practitioner-facing reports and consulting deliverables.
- Asking what the community gains from this research, and building the community benefit into the dissemination plan from the start.

### Epistemic justice as practical infrastructure

The case for epistemic justice in neurodiversity research and practice rests on both the ethical principles and on an empirical observation: knowledge built with communities is likely to be more accurate, more useful, and more durable than knowledge built about them (Mottron et al., 2006; Mottron, 2011; Pellicano and den Houting, 2022; Pickard et al., 2022; Praslova, 2024). When neurodivergent people are treated as knowers and the foremost experts on their own experiences, strengths, and workplace needs, science gains access to insights that less participatory models cannot replicate.

Neuroinclusive, evidence-based practices that support individual, organizational, and community flourishing must begin with knowledge that accurately reflects neurodivergent experiences. Epistemic justice makes that accuracy possible while also upholding human dignity (Hicks, 2011) and moral principles of respect for autonomy, non-maleficence, beneficence, and justice in research and practice (Beauchamp and Childress, 2019).

## Data Availability

The original contributions presented in this study are included in this article/supplementary material, further inquiries can be directed to the corresponding author.
